# DNA maintenance in plastids and mitochondria of plants

**DOI:** 10.3389/fpls.2015.00883

**Published:** 2015-10-29

**Authors:** Delene J. Oldenburg, Arnold J. Bendich

**Affiliations:** Department of Biology, University of Washington, Seattle, WA, USA

**Keywords:** chloroplast DNA, DNA recombination, DNA repair, DNA replication, organellar DNA

## Abstract

The DNA molecules in plastids and mitochondria of plants have been studied for over 40 years. Here, we review the data on the circular or linear form, replication, repair, and persistence of the organellar DNA (orgDNA) in plants. The bacterial origin of orgDNA appears to have profoundly influenced ideas about the properties of chromosomal DNA molecules in these organelles to the point of dismissing data inconsistent with ideas from the 1970s. When found at all, circular genome-sized molecules comprise a few percent of orgDNA. In cells active in orgDNA replication, most orgDNA is found as linear and branched-linear forms larger than the size of the genome, likely a consequence of a virus-like DNA replication mechanism. In contrast to the stable chromosomal DNA molecules in bacteria and the plant nucleus, the molecular integrity of orgDNA declines during leaf development at a rate that varies among plant species. This decline is attributed to degradation of damaged-but-not-repaired molecules, with a proposed repair cost-saving benefit most evident in grasses. All orgDNA maintenance activities are proposed to occur on the nucleoid tethered to organellar membranes by developmentally-regulated proteins.

## Introduction

In diploid plants and animals, the chromosomes of both parents are present in the nuclei of nearly all cells. Replication precisely duplicates the chromosomal DNA molecules, and checkpoint control ensures partition of the duplicated chromosomes to daughter cells only after all DNA damage is repaired, leading to constant properties of chromosomal DNA among tissues during development from embryo to adult. In contrast, the properties of chromosomal DNA molecules in the plastids and mitochondria change drastically during development. Why are organellar chromosomes not constant in cells containing constant nuclear chromosomes, despite the fact that the replication/repair apparatus for all cellular DNAs is encoded exclusively by the nuclear genome? The principal reason, we suspect, is that the level of DNA damage is far greater in the organelles than the nucleus. Furthermore, if an organellar DNA molecule is damaged but not repaired, the DNA molecule carrying the damage will be degraded in order to prevent mutagenesis—DNA abandonment (Bendich, 2010b, 2013). In this article, we describe the replication, repair, and persistence of chromosomal DNA molecules in plastids and mitochondria. We conclude that whereas DNA repair suffices for the nucleus, organellar DNA (orgDNA) turnover, copy number change, and abandonment are also needed to maintain cellular homeostasis during development.

## The Size and Structure of Organellar DNA Molecules in Plants: A Historical Perspective

In 1963, an autoradiographic image of an evidently intact DNA molecule from a lysed cell of *Escherchia coli* strain K12 was published at a time before the genome size of *E. coli* was known (Cairns, 1963). This molecule had the form of the Greek letter “theta,” had no ends, and appeared to be undergoing replication. This single example reported of such a theta molecule gave rise to the notion that the bacterial genome was carried on one circular chromosome and profoundly influenced future research on the size and form of DNA molecules from chloroplasts and mitochondria. The measured length of each of the loops of the theta (the replicated portion of the molecule), when added to that of the unreplicated portion, gave a total of 1100 microns. Subsequent work with *E. coli* K12 revealed that the genome size was 4.6 Mb, equivalent to 1580 microns of B-form DNA, most circular molecules were much smaller than genome size (with a few at 2000–4000 microns), and circular molecules were extremely infrequent among all molecules (reviewed by Bendich, 2007). Nonetheless, the expectation was created that chromosomal DNA molecules in plastids and mitochondria would be found on genome-sized circular molecules, as in their bacterial ancestors, and this expectation is still widely held. For the mitochondrial genome of yeast, it took more than 30 years to realize that the nearly exclusively non-circular forms of mitochondrial DNA (mtDNA) should not have been dismissed as “broken circles,” but actually represented the wild-type chromosomes (Williamson, 2002; Bendich, 2007, 2010b).

**FIGURE 1:**
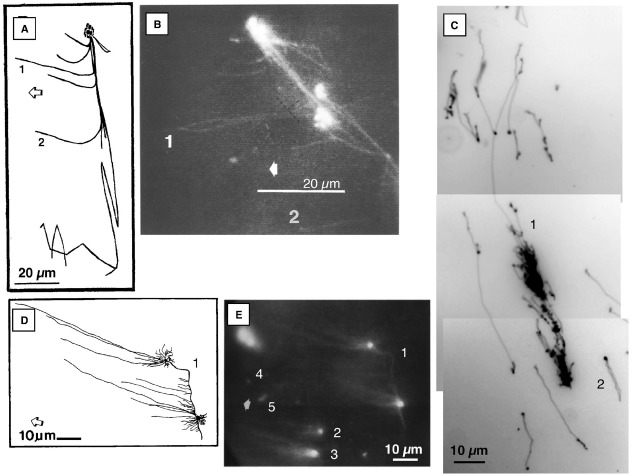
**Fluorescence microscopic images of ethidium-stained mtDNA and ptDNA molecules. (A)** and **(B)** Images of DNA-protein structure from osmotically lysed tobacco BY-2 mitochondria. Complex branching DNA-protein structure with three bright nodes, long immobile fiber, and several fibers that extend leftward toward the anode (examples: 1 and 2) and rightward when the polarity of the electric field was reversed. (Adapted from Oldenburg and Bendich, 1998b). **(C)** Maize ptDNA molecules from the well-bound fraction following PFGE. Examples: (1) multigenomic complex structure with a Y-branch and (2) a genome-sized circular molecule. Approximately 84% of the DNA mass was in the large complex form, 11% in small branched molecules, and 4% in circular molecules. The in-gel ptDNA was prepared from 14-day maize seedlings. (Adapted from Oldenburg and Bendich, 2004a) **(D)** and **(E)** Images of liverwort mtDNA molecules from the well-bound fraction following PFGE. One large complex structure with two bright nodes of fluorescence that are connected by a bright fiber and several fibers extend from each node toward the anode (1). Two smaller “comet” structures with several “tail” fibers extending from the bright ”head” (2, 3). A few small molecules were moving toward the anode (examples: 4, 5). (Adapted from Oldenburg and Bendich, 1998a) The molecules in **(B)** and **(E)** were recorded using an epifluorescence microscope equipped with a CCD camera, video monitor, and recorder. Photographs were then taken of ethidium-stained DNA on the monitor and the respective drawings, **(A)** and **(D)**, were made by tracing the DNA on the monitor. The molecules in **(C)** were recorded using an epifluorescence microscope equipped with a digital camera and computer. Broad arrows point toward the anode in **(A)**, **(B)**, **(D)**, and **(E)**.

For plants, contaminating nuclear DNA was successfully removed from mtDNA (Kolodner and Tewari, 1972a) and plastid DNA (ptDNA; Kolodner and Tewari, 1972b) in pea. Electron microscopy (EM) and DNA reassociation kinetics analysis (DRKA) led to the conclusion that the chromosomes in both organelles were present as genome-sized circles. For the chloroplasts, however, the DNA was fractionated before EM, which probably removed the very large and branched molecules subsequently revealed in total DNA obtained from plastids. In-gel procedures, pulsed-field gel electrophoresis (PFGE), and moving pictures of ethidium-stained molecules (DNA Movies) showed circular ptDNA as a minor component with most ptDNA in simple linear and branched forms (Figure 1C). For the mtDNA, some circular forms were of a size also obtained from DRKA, but this size is much smaller than the genome size of the pea mitochondrial genome subsequently obtained by DRKA and restriction fragment summation.

## Chromosomal DNA Molecules in the Mitochondria and Chloroplasts of Plants: Circular or Linear?

In 1972 the chromosomes in plant mitochondria and chloroplasts were proposed to exist as genome-sized circular DNA molecules (Kolodner and Tewari, 1972a,b). Considering the profound influence of this conclusion on subsequent research, it is instructive to review the original evidence for circular chromosomes in plant mitochondria and chloroplasts.

Using EM with the contents released from osmotically-shocked mitochondria, 55% of the circular molecules that were measured were in circular form and ∼30 microns (87 kb) in contour length, “10% of the circular molecules were present in dimer-length circles” (63–64 microns), and the longest linear mtDNA reported was 51 microns (Kolodner and Tewari, 1972a). After treatment with protease and chloroform, 25% of the mtDNA was found as circles of about 30 microns. The authors concluded that molecules of DNA in pea mitochondria are circular with a molecular weight of about 70 Md (106 kb).

Kolodner and Tewari (1972a) also estimated the size of the pea mitochondrial genome from the rate with which denatured mtDNA strands reassociated relative to that for T4 phage DNA (size of 106 Md): 0.70 × 106 = 74 Md or 112 kb, using 662 d per base pair. This value becomes 190 kb when the apparent kinetic complexity of 180 Md (272 kb) for glucosylated T4 DNA is used (Ward et al., 1981). From DRKA, the size of the mitochondrial genome in pea was estimated by Ward et al. (1981) as ∼215 Md (325 kb) when the genome size of the *Bacillus subtilis* standard was taken as 3500 kb. Using 4200 kb for the *B. subtilis* genome from sequencing data, Alverson et al. (2010) employed a 1.2-fold correction, which gives 390 kb for the pea mitochondrial genome. The size of the pea mitochondrial genome obtained by summing the lengths of restriction fragments was ∼320 (Ward et al., 1981) and 430 kb (Stern and Palmer, 1984). To our knowledge, there are no genome size estimates from mitochondrial genome sequencing for pea. These data show that the 87-kb class of circular mtDNA molecules found by EM represents circles of subgenomic size, rather than circles of genome size as inferred by Kolodner and Tewari (1972a).

The size of DNA molecules from pea chloroplasts was measured by EM (Kolodner and Tewari, 1972a). The circular molecules, which accounted for as much as 37% of all measured DNA length, were tightly grouped near 39 microns (115 kb), and “none of the linear molecules…were…longer than the length of the circular molecules.” Using 106 Md for the T4 DNA standard, the kinetic complexity was reported as 94.6 Md (143 kb), which becomes 243 kb after correcting for T4 DNA glycosylation. The size of the pea plastid genome is 120 kb from restriction fragment summation (Palmer and Thompson, 1981) and 122 kb from genome sequencing (accession NC_014057).

These data show that the circular ptDNA molecules reported by Kolodner and Tewari (1972a,b) closely approximate the genome size as determined from restriction fragments and genome sequencing, whereas their DRKA data do not closely approximate the EM data or the genome size. How can we reconcile the EM data showing no linear molecules larger than the genome-sized circles with the data from PFGE and DNA Movies showing much or most of the ptDNA from pea and other plants in linear and branched-linear forms larger than the size of the genome (Bendich and Smith, 1990; Oldenburg and Bendich, 2004a; Shaver et al., 2006)? For PFGE and DNA Movies, the procedure starts with plastids embedded in agarose (in-gel), so that none of the DNA present in the organelles can be removed before analysis. Most of the in-liquid procedures described for both ptDNA and mtDNA, include centrifugation at 12,000 × g for 30 min before the supernatant is sampled for analysis by EM (Kolodner and Tewari, 1972a,b). We suspect that the large, complex forms of orgDNA would be removed by this centrifugation, so that the orgDNA was fractionated prior to analysis. Furthermore, any complex, branched molecules seen by EM might have been deemed uninterpretable and excluded from analysis. A hint that this may have occurred is that “lysed preparations…often resulted in tangled molecules” (Kolodner and Tewari, 1972b). In one of the procedures, osmotic shock was used to release the contents of isolated mitochondria, and EM was conducted without prior fractionation by centrifugation (Kolodner and Tewari, 1972a). When isolated mitochondria from tobacco and yeast were first embedded in agarose and then subjected to hypotonic medium to cause lysis within the gel, subsequent DNA Movies revealed apparently “naked” DNA molecules as well as enormous, complex, branched forms that migrated to the cathode during electrophoresis, indicating that they were bound to basic proteins (Figures 1A,B; Oldenburg and Bendich, 1998b). We suspect that such complex forms would have been present in the lysed preparations produced by osmotic shock of pea mitochondria, but may have been dismissed as uninterpretable tangled molecules.

In conclusion, circular forms of orgDNA from plants appear to have exerted a profound influence on 40 years of research, despite the weakness of the data in support of the notion that most or all functions of organellar chromosomes are served by circular DNA molecules (Williamson, 2002; Bendich, 2010a). When in-gel methods are employed, chromosomal DNA molecules in the plastids and mitochondria of plants appear as linear and branched-linear forms of various sizes (Figure 1), are found in meristematic tissues, and are typically larger than the size of the genome. In maize, tobacco, and *Medicago truncatula*, restriction digest analysis showed that the linear molecules have ends at defined regions of the plastid genome and isomers with three to six distinct ends (Oldenburg and Bendich, 2004a; Scharff and Koop, 2006, 2007; Shaver et al., 2008). For maize, the precise locations for two ends have been determined by sequencing, and both are near putative origins of replication (Oldenburg and Bendich, unpublished results). The circular forms account for a few percent or less of total orgDNA (Bendich, 1996; Oldenburg and Bendich, 1996, 1998a, 2004a) and are proposed to represent defective forms of orgDNA akin to the circular mtDNA molecules in *petite* mutants of yeast (Bendich, 2010a).

## Copy Number and Integrity of Organellar Genomes during Plant Development

One of the curious facts about orgDNA in plants is that the number of genome equivalents (hereafter termed “copy number”) per diploid cell is large and highly variable during plant development, whereas the copy number in the nucleus of the diploid cell remains essentially constant throughout development. The curiosity of this fact increases when one considers that orgDNA-encoded proteins persist at fixed molar ratios with their nuclear DNA-encoded subunit partners in multi-subunit protein complexes, such as ribosomes, cytochrome oxidase, and RUBISCO. These facts alone indicate that the strategy for regulating gene expression differs greatly between the nuclear and organellar genomes. In an early proposal, high copy number of orgDNA reflects an increased demand for organellar ribosomes that can only be satisfied by increased rRNA gene number that results from genome amplification (Bendich, 1987). Recently, additional insight was obtained from the concept of DNA abandonment in which some or all of the copies of orgDNA, but not nuclear DNA, can be degraded during development because they have served their coding function and are damaged but not repaired (Bendich, 2010b, 2013).

Several methods have been used to estimate orgDNA copy number in plants: (i) measuring the increase in the rate of probe DNA strand reassociation caused by the addition of a large amount of DNA extracted from total tissue (Lamppa and Bendich, 1979a, 1984); (ii) blot hybridization of a probe to restriction-digested total tissue DNA (Li et al., 2006; Zheng et al., 2011; Udy et al., 2012; Oldenburg et al., 2013); (iii) fractionation of orgDNA by PFGE (Oldenburg et al., 2006; Shaver et al., 2006; Oldenburg et al., 2013); (iv) quantitative fluorescence using a DNA-specific fluorophore and either intact cells or organelles isolated from cells (Oldenburg and Bendich, 2004b; Rowan et al., 2004; Shaver et al., 2006; Oldenburg et al., 2013); and (v) real-time quantitative PCR (qPCR; Zoschke et al., 2007; Rowan et al., 2009; Preuten et al., 2010; Udy et al., 2012). These procedures should yield equivalent results providing that the molecular integrity of the DNA molecules is maintained, as is the case for chromosomal DNA in the nucleus.

Molecular integrity, however, changes drastically during development. The most sensitive assay we have to monitor molecular integrity is the analysis of DNA molecules using DNA Movies. Isolated organelles are first embedded in agarose gel (as in preparation for PFGE), the gel is soaked in detergent, EDTA, and proteinase K to release intact DNA, and the movement of ethidium-stained molecules with and without an electric field in real time can be observed and recorded. Circular molecules up to several megabases in size are clearly distinguished from linear and branched forms, lengths of individual molecules can be measured, and a single double-strand break can be monitored (Bendich, 1991, 1996, 2001). PFGE (method iii) is also highly sensitive, and quantitative fluorescence (method iv) less so, to a decrease in molecular integrity, whereas methods i, ii, and v measure molecules fragmented either intentionally (by shearing or restriction digestion) or within the plant cell (by DNA damage response activities; see below). Recently, a method was developed for conducting quantitative PCR using primers spaced by 11 kb (long-PCR), rather than the typical spacing of about 0.1–0.15 kb used for qPCR: this is method (vi) molecular integrity PCR (miPCR), and orgDNA copy numbers were determined with both qPCR and miPCR during development of maize seedlings (Kumar et al., 2014, 2015). DNA copy number values using standard qPCR exceeded those using miPCR by 100-to 1000-fold, with the greatest disparity found for light-grown leaves. The drastic decrease in orgDNA molecular integrity from multigenomic structures in the meristem to less-than-genome-sized fragments in green leaf tissues revealed using DNA Movies and PFGE is paralleled in copy number assays using miPCR but not standard qPCR. Mechanistically, there is at least one single- or double-strand break or DNA polymerase-blocking lesion per 11 kb in nearly every molecule of orgDNA in green leaf, but such impediments to DNA amplification are infrequent at a length of 0.15 kb. In other words, orgDNA in green leaf tissue has been damaged, not repaired, and degraded to the small fragments detected in DNA Movies and the smear at the bottom of the gel in PFGE. Furthermore, about one-third of the ptDNA from green leaf is so small that it diffuses out of the gel plugs during the post-lysis plug washes and is lost before PGFE analysis begins (Kumar et al., 2014), whereas these fragments would still be scored as “copies” using standard qPCR and total tissue DNA. We conclude that the measurement of orgDNA copy number depends strongly on the assay method. Estimates provided by qPCR are probably accurate for meristematic cells containing the multigenomic molecules revealed by PFGE and DNA Movies, but greatly overestimate the level of functional DNA in mature leaves.

**FIGURE 2:**
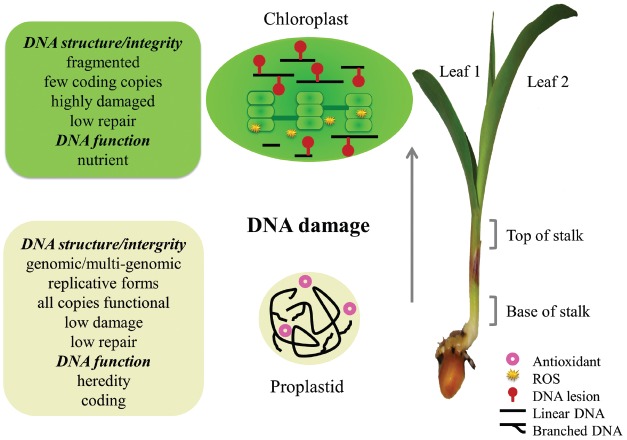
**Changes in orgDNA during maize development.** Recombination-dependent replication of orgDNA in the basal meristem produces branched, multigenomic chromosomes in proplastids and mitochondria (not depicted). DNA-damaging oxidative stress is minimized, requiring little repair, by maintaining hypoxia, antioxidants, and no ROS-generating photosynthesis or respiration. Early in leaf development, orgDNA damage occurs due to ROS generated in photosynthesis, respiration, and oxidation of pigments and lipids. Later, when the damage level exceeds the repair capacity, orgDNA is fragmented and no longer functions in coding or heredity, mitochondria switch from respiration to photorespiration, and DNA copy number declines faster for mitochondria than for chloroplasts. (Reprinted from Kumar et al., 2014).

What causes intact orgDNA to become highly degraded when cells from the shoot meristem develop into green leaf cells? A damaged-but-unrepaired molecule is typically degraded in bacteria (Skarstad and Boye, 1993) and human mitochondria (Shokolenko et al., 2009), thus avoiding mutation, and we suggest the same applies to plant orgDNA (Figure 2). In consequence, almost none of the “copies” scored by standard qPCR for a green maize leaf serve a coding function. Since the rate of orgDNA decline during leaf development differs among plant species, with maize being the most extreme example among the five plants investigated (Shaver et al., 2006; Rowan and Bendich, 2009; Oldenburg et al., 2014), the transition from coding to nutrient function for orgDNA is expected to occur at different rates among plants. For example, the brightness of DAPI-stained plastid nucleoids decreased with age of the leaves over a 4-year period on a single branch of an evergreen Rhododendron shrub; there were no 5-year-old leaves (Oldenburg and Bendich, unpublished results).

## Proteins Associated with Organellar DNA Replication, Recombination, and Repair

Proteins involved in orgDNA replication, recombination, and repair have been identified, the activities of a few have been investigated genetically, and changes in the levels of proteins found in plastids and mitochondria during leaf development have been revealed by proteomic analysis (Marechal and Brisson, 2010; Krupinska et al., 2013; Cupp and Nielsen, 2014; Moriyama and Sato, 2014; Powikrowska et al., 2014). These proteins are nuclear-encoded and can be categorized according to function and organelle localization. We now focus on the relationship between these proteins and orgDNA quantity/quality as the leaf develops.

Most orgDNA-associated proteins are largely confined to the meristem (proplastids) and young leaves (developing chloroplasts). For example, the DNA polymerase, PolIA, was found in proplastids but not chloroplasts of maize (Majeran et al., 2012), and the plastid DNA polymerase genes of *Arabidopsis* and rice were expressed in meristematic tissues, not in mature green leaves (Kimura et al., 2002; Mori et al., 2005). Expression as determined by qRT-PCR of the *Arabidopsis* gene for the helicase/primase TWINKLE was greatest in young leaves and shoot apex tissues, and its protein level was shown to decrease with increasing age of rosette leaves (Diray-Arce et al., 2013).

Some DNA proteins are dual-targeted to both plastids and mitochondria, including PolIA, PolIB, Twinkle, and the recombination protein RecA2. Most proteins known to be dual-targeted are associated with DNA maintenance and mRNA translation (Carrie and Small, 2013). And yet, for some DNA-associated proteins there are plastid-specific and mitochondrial-specific homologs. For example, Why1, Why3, and RecA1 are plastid-targeted, whereas Why2 and RecA3 are mitochondria-targeted. Is there a functional explanation for the persistence of both dual-targeted and organelle-specific DNA maintenance proteins? We proposed that during development there is a shift in a major mitochondrial function, from respiration to photorespiration, that is coordinated with the transition of non-green plastids to photosynthetically-active chloroplasts (Oldenburg et al., 2013; Kumar et al., 2014). Examples of tissue- or cell type-specific differences that would require such coordination are: roots compared with green leaves; and meristematic cells compared with mesophyll or epidermal cells. One way to achieve the coordination is to produce dual-targeted proteins such as those in the replisome, whereas the organelle-specific proteins would be useful for modulating the amount of functional (undegraded) orgDNA in a tissue-specific manner. Mitochondrial and plastid functions may thus coordinately respond to signals such as the redox state of the cell (Millar et al., 2011). For example, in meristematic cells conducting “quiet” metabolism [no respiration, no photosynthesis, low reactive oxygen species (ROS), Bendich, 2010b], dual-targeting of replisome proteins would maintain the copy number of both mtDNA and ptDNA at the pre-differentiation copy number. However, in roots where respiration is required, higher levels of mtDNA would be retained than in green mesophyll cells where the primary mitochondrial function is photorespiration. Thus the organelle-specific proteins may determine the selective retention or degradation of orgDNA among tissues. During development and upon receipt of the light-dependent phytochrome signal, cellular differentiation begins, the cellular redox state changes, and plastid-specific and mitochondrial-specific proteins would exert their effects on orgDNA levels and integrity in a tissue-specific manner (Zheng et al., 2011; Oldenburg et al., 2013; Kumar et al., 2014).

As the synthesis of proteins used during photosynthesis increases, the production of additional DNA to meet the increasing demand for gene products might be expected to increase, with the highly-labile D1 protein (the *psbA* gene product) as a critical example. Yet, as discussed above, the abundance of the proteins needed to produce and maintain ptDNA actually decreases. This observation is consistent with the declining copy number of ptDNA during leaf development and the high stability of psbA mRNA, but unexpected under the hypothesis that the fully functional gene for D1 must persist in mature maize leaves.

If orgDNA is to be maintained, replication/repair proteins should be present and active in these organelles, as in single-celled organisms like yeast, *Chlamydomonas*, and *Euglena*, and the cells leading to the germ cells of plants and animals. An alternative is to abandon orgDNA in somatic cells by not supplying those proteins. The proteomic analysis indicates that during leaf development in maize the level of replication/repair proteins targeted to chloroplasts decreases relative to proplastids. Although the activity of these proteins was not addressed, this decrease in the levels of orgDNA maintenance proteins is consistent with orgDNA abandonment in maize.

## Organellar Nucleoids: Where the Action is

After staining with a DNA fluorophore, brightly fluorescing regions within plastids and mitochondria identify regions that contain high concentrations of DNA: the nucleoids. Nucleoids *in situ* appear in various forms, including dots that may or may not be connected by fibers and may be located at the periphery or toward the interior of the plastid (Coleman, 1979; Kuroiwa et al., 1981). The size and fluorescence intensity of the nucleoid reflect the DNA content, which can vary enormously among plant cells (Kuroiwa et al., 1981; Kuroiwa, 1991). When isolated from the organelles, nucleoids are found to contain DNA, RNA, and proteins, including the plastid-encoded RNA polymerase in the “transcriptionally active chromosome” (reviewed in Krupinska et al., 2013; Liere and Börner, 2013). It is believed that the functions of orgDNA (inheritance, replication, repair, and transcription) are served largely or exclusively from nucleoids bound to membranes (Gilkerson et al., 2013; Kindgren and Strand, 2015). We now combine morphological and biochemical data for nucleoids to elucidate the process of orgDNA maintenance during plant development.

An early study in tobacco showed that the composition of nucleoid-associated proteins differed between proplastids and chloroplasts (Nemoto et al., 1990). The nucleoids of maize plastids contain proteins associated not only with DNA, but also RNA metabolism including transcription, mRNA processing, and stability (Majeran et al., 2012). Changes in RNA-associated proteins indicated transcription as the primary function in developing plastids and mRNA translation and protein homeostasis in chloroplasts. Although many nucleoid-enriched proteins were assigned a function, function was not assigned to many others, including PPR proteins (likely associated with RNA processes). Of the DNA-associated nucleoid proteins (including those for replication/repair and ROS protection), most were more abundant in proplastids than chloroplasts, with the exception of three DNA repair proteins that were more abundant in the tip than the base of the leaf (Majeran et al., 2012). Two of these (FAD photolyase and a uvrB/uvrC-motif protein) likely function in repair of UV-induced damage and the third (MutS2) may function to suppress illegitimate recombination (Kang et al., 2005; Pinto et al., 2005; Fukui et al., 2007), so that none of these three is likely associated with repair of ROS-induced DNA damage. The primary repair pathway for ROS-induced oxidative lesions is base excision repair (BER), and in *Arabidopsis* BER enzymes were found in both mitochondrial and plastid nucleoids (Gutman and Niyogi, 2009; Boesch et al., 2011), although no information was given about the stage of plastid developmental or enzyme abundance.

A DNA-membrane anchoring function has been assigned to some nucleoid proteins, such as PEND specific for the plastid envelope and MFP1 for the thylakoids (Krupinska et al., 2013; Liere and Börner, 2013). In maize, six such anchoring proteins were identified, although only three were enriched in isolated nucleoids and one of these (pTAC16) was enriched in the leaf tip relative to the base (Majeran et al., 2012). Several proteins were classified as “DNA organization and quality control” (such as YlmG1 and Why1; Majeran et al., 2012) that may also mediate membrane attachment either directly or indirectly through protein-protein interactions. A function in nucleoid partitioning was reported for the YlmG1 family of proteins (Kabeya et al., 2010), and in maize YlmG1-1 was enriched in proplastids whereas YlmG1-2 was enriched in chloroplasts (Majeran et al., 2012). The single-strand DNA-binding Whirly proteins are associated with nucleoids in plastids and mitochondria (Prikryl et al., 2008; Marechal and Brisson, 2010), and Why1 in maize is more abundant in proplastids than chloroplasts (Table 1 in Majeran et al., 2012). Thus during plastid development, changes in nucleoid protein composition likely reflect changes in DNA-membrane attachment.

Although the various forms (dots, rings, fibers) and plastid locations (peripheral, central, scattered) of plastid nucleoids were originally considered as characteristic for the plant or algal group, these morphological properties were found to change during proplastid-to-chloroplast development in wheat and *Arabidopsis* (Miyamura et al., 1986; Fujie et al., 1994). In proplastids and developing plastids, nucleoids are attached to the envelope membrane whereas in chloroplasts the nucleoids are attached to the thylakoid membrane (Krupinska et al., 2013; Powikrowska et al., 2014). Combined with the changing protein composition during plastid development, it now seems likely that nucleoid appearance *in situ* reflects the biochemical activity of the cell. Attachment of orgDNA molecules to membranes *in vivo* would affect their maintenance, according to the following scenario. We suggest that (1) the three activities maintaining DNA integrity—replication, recombination, and repair—take place only on DNA firmly associated with membrane-attached nucleoids; (2) changes in nucleoid protein composition during development can result in release of damaged-but-unrepaired DNA from the membrane/nucleoid; (3) this unbound DNA is now susceptible to further degradation by nucleases; and (4) this process is indicated by the decrease in nucleoid size and DAPI-DNA intensity and ultimately the complete disappearance of nucleoids in many mature chloroplasts of maize.

If replication/repair requires that a DNA end be attached to the membrane, then once the DNA molecule leaves the membrane it can no longer replicate or be repaired and would be degraded by exonucleases. A supercoiled circular DNA has no end, cannot be replicated—and pulse-labeling shows it is not first-labeled—and leaves the membrane. But it would not be digested by exonucleases and could still be detected by EM, PFGE, and (in relaxed circular form) DNA Movies and be enriched in the supernatant after high-speed centrifugation (which would pellet the large complex forms), as performed by Kolodner and Tewari (1972a,b). The circular forms account for a few percent or less of total orgDNA, are proposed by-products of recombination used to replicate linear DNA (Bendich, 1996; Oldenburg and Bendich, 1996, 2004b), and are unlikely to serve as templates for DNA replication/repair or transcription within the organelles. Mung bean mtDNA was analyzed both from entire mitochondria and from nucleoids isolated from the mitochondria. For nucleoids, >50% of the mtDNA molecules were found in complex forms and ∼30% were linear by EM; well-bound and 50–200-kb fractions were found by PFGE (Lo et al., 2011). For entire mitochondria, an additional prominent fraction was found at <50 kb (Dai et al., 2005), which we suggest was not associated with the nucleoid-on-membrane and resulted from nuclease digestion *in vivo*.

## The Replication of Organellar DNA in Plants

The first model for the replication of plant orgDNA was proposed for ptDNA by Kolodner and Tewari (1975) and was based exclusively on EM images: circular products from a circular template involving a displacement loop and theta-type replication. Subsequently, ^3^H-labeled thymidine was used in pulse-chase experiments with cultured tobacco cells to quantify the forms of replicating mtDNA fractionated by PFGE (Oldenburg and Bendich, 1996). The first-labeled form was found in the well-bound fraction of the gel, with a zone of linear molecules at about 50–150 kb accumulating the tritium with time at the expense of the well-bound form. Genome-sized molecules (430 kb for tobacco; Sugiyama et al., 2005) in either linear or circular form were not detected by analysis of either radioactivity or ethidium staining. A well-bound precursor and a 50–200-kb product were also shown for mtDNA synthesis in mung bean seedlings (Dai et al., 2005). For cultured liverwort cells, the well-bound fraction, not the circular genome-sized band (≤5% of all mtDNA), contained the earliest form(s) of mtDNA produced during replication. The well-bound DNA is immobile during PFGE because of its large size and complex branching form (Figures 1D,E; Oldenburg and Bendich, 1998a).

Using a cytological approach and incorporation of bromodeoxyuridine (BrdU) to monitor DNA synthesis in roots of *Pelargonium* and *Arabidopsis* seedlings, most mtDNA synthesis was found in mitochondrial nucleoids of enormous size (several megabases of mtDNA) in the root tip meristem, with nucleoids containing ∼90–140 kb of mtDNA in the root elongation zone (Kuroiwa et al., 1992; Fujie et al., 1993). Since the mitochondrial genome size is 367 kb for *Arabidopsis* (Unseld et al., 1997) and likely to be much larger than 140 kb for *Pelargonium*, the general conclusion is that replication of plant mtDNA occurs in meristematic cells with molecules of multigenomic size that are converted to simple linear forms of about 50 to 200 kb in non-dividing cells that no longer replicate their mtDNA. The same cytological/BrdU procedures were used to identify meristematic cells as the principal or only cell type in which ptDNA was replicated in roots of *Arabidopsis* and rice (Fujie et al., 1993; Suzuki et al., 1995). In maize, mtDNA replication was highest in the metabolically-active embryo and was also found in both roots and stalk, but not in the mature leaf blade (Oldenburg et al., 2013). As the first foliage leaf of *Arabidopsis* developed, the number of genomes per plastid increased from ∼40 (3 days after seeds were sown) to 600 at day 7, when the leaf was <0.5 mm in length, whereas genome equivalents per mitochondrion decreased from 2 to <0.5 during this interval (Fujie et al., 1993, 1994). Similarly, in maize and other cereals, ptDNA replication was most intense in the stalk region above the basal meristem (Baumgartner et al., 1989; Hashimoto and Possingham, 1989; Oldenburg et al., 2006; Zheng et al., 2011). The replication of ptDNA in maize is stimulated by light, although it also occurs in dark-grown seedlings (Oldenburg et al., 2006; Zheng et al., 2011) and in the dark for *Chlamydomonas* growing on acetate (Kabeya and Miyagishima, 2013). *Chlamydomonas* ptDNA replication is regulated by the cellular redox state (Kabeya and Miyagishima, 2013).

Regions of the plastid genome that best supported DNA synthesis *in vitro* were designated as replication origins (*oris*), and led to the assignment of two major *oris* (known as oriA and oriB) in *Oenothera*, tobacco, and pea (Heinhorst and Cannon, 1993; Kunnimalaiyaan and Nielsen, 1997). Sequences similar to those of oriA and oriB have been identified in the plastid genomes of many plants (Oldenburg and Bendich, 2004a; Shaver et al., 2008; Krishnan and Rao, 2009). Plastid origin-binding proteins (OBP) have been identified for *Chlamydomonas* (Nie et al., 1987) and soybean (Lassen et al., 2011).

Three types of replication mechanism have been proposed for ptDNA: theta replication, rolling circle replication (RCR), and recombination-dependent replication (RDR; Kunnimalaiyaan and Nielsen, 1997; Marechal and Brisson, 2010). Although circular ptDNA molecules were reported for chloroplasts from entire light-grown shoots of several plants (Kolodner and Tewari, 1972b; Lamppa and Bendich, 1979b; Bendich and Smith, 1990; Lilly et al., 2001), support for the theta and RCR mechanisms would seem to require the presence of circular ptDNA molecules in meristematic tissues. The base of the leaf in grasses is a rich source of meristematic cells. Using blot-hybridization and PFGE fractionation, a sharp band representing a supercoiled circular form of ptDNA (but only 3% of all ptDNA) was detected in dark-grown first and second leaf blade, but not stalk (meristem at the base of the leaf) tissue of maize seedlings. However, no circular ptDNA was detected in light-grown stalk, and in light-grown leaf blade most of the ptDNA was found as less than-genome-sized fragments and often barely detectable (Oldenburg and Bendich, 2004b; Oldenburg et al., 2006). Thus, support was not obtained for the theta or RCR models in maize. In fact, light triggered the rapid degradation of all forms of ptDNA (Oldenburg et al., 2006; Zheng et al., 2011). The circular ptDNA was found in a tissue no longer engaged in ptDNA replication. Support for RDR would seem to require the presence of multigenomic, branched molecules in the meristem. For stalk tissue, the well-bound fraction contained a large amount of ptDNA, and most ethidium-stained molecules imaged by fluorescence microscopy were in complex branched forms (Figure 1C; Oldenburg and Bendich, 2004b), in support of the RDR mechanism. These complex forms were also found in young leaf tissue of *Arabidopsis*, tobacco, and *Medicago truncatula* (Rowan et al., 2004; Shaver et al., 2006).

To summarize, we know rather little of the details of orgDNA replication in plants. The evidence indicates, however, that circular forms of the plastid genome, while detectable in some plant tissues, are not the principal template for ptDNA replication, and circular forms of the entire mitochondrial genome—the “master circle”—have been reported only for cultured liverwort cells. The data we do have are compatible with linear DNA molecules and an RDR mechanism for both mtDNA and ptDNA in which multiply-branched molecules larger than the size of the genome provide the orgDNA for progeny cells. Given the paucity of mutants with which to investigate orgDNA replication in plants, we may draw mechanistic inference from other DNA replication systems and data from organellar proteomics. For example, structural similarities between ptDNA and herpes simplex virus (HSV) DNA include a linear genome of ∼150 kb, two single-copy regions separated by inverted repeats (IRs), and multigenomic branched-linear replicative forms. Furthermore, although a theta-to-rolling-circle model was initially suggested, a RDR mechanism with linear molecules is now proposed for the replication of HSV DNA (Weller and Sawitzke, 2014).

Let us consider three processes associated with DNA replication: (1) initiation and opening of the double helix; (2) loading replication proteins and establishment of a replication fork; and (3) single-strand annealing (SSA) and recombination. In HSV DNA there are three *oris*, one in the long single copy region (U_*L*_) and two in the IRs. Initiation occurs when UL9 (an OBP) binds to an *ori* leading to recruitment of the replisome (helicase/primase, DNA polymerase, etc.), followed by opening of an adjacent A/T-rich region and formation of a replication fork with both leading- and lagging-strand synthesis (Weller and Sawitzke, 2014). Similarly, a plastid OBP could bind at oriA/oriB (Lassen et al., 2011) recruiting the plastid replisome (Twinkle, PolIA, etc.; Moriyama and Sato, 2014). Although the OBP/*ori* system is widely used to initiate DNA replication, initiation could also occur by transcription, specifically in the rRNA genic region. Plastid *oris* are located near the rRNA genes in many organisms, leading to a transcription-coupled DNA replication process whereby transcription-mediated helix opening could allow subsequent access of the replisome (Chang and Wu, 2000).

**FIGURE 3:**
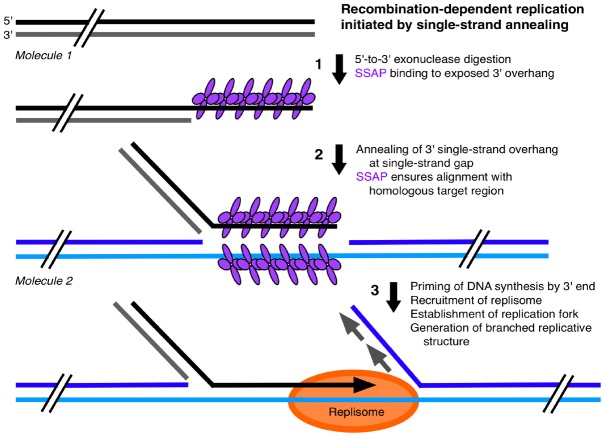
**Single-strand annealing mechanism for plastid DNA replication.** This single-strand annealing (SSA), recombination-dependent replication model for ptDNA is based on a replication mechanism for herpes virus DNA (Weller and Sawitzke, 2014). **(1)** A 3′-overhang is generated by 5′-to-3′ exonuclease digestion at the end of a unit-genome-sized monomer. A single-strand annealing protein (SSAP) binds to a 3′-overhang. **(2)** Annealing of the 3′-overhang of *Molecule 1* to a homologous single-strand gap in another ptDNA molecule (*Molecule 2*). **(3)** Replication is initiated by priming at the 3′-end, assembly of the replisome, and formation of a replication fork, leading to a branched-linear structure. A similar model with the same or analogous proteins applies to the replication of mtDNA in plants. We propose that replication occurs only with ptDNA attached to the nucleoid-on-membrane using single-strand end-binding proteins. Although we propose that Whirly proteins serve attachment and SSAP functions, other single-strand-binding proteins, such as the OSB and RecA families, may also participate in ptDNA replication. Other replication and recombination mechanisms have been described (Cox, 2007; Marechal and Brisson, 2010; Weller and Sawitzke, 2014; Morrical, 2015).

A SSA mechanism has been described for HSV DNA that can generate concatemers, initiate DNA synthesis, and produce branched replicative forms (Weller and Sawitzke, 2014). We propose an analogous SSA mechanism for plant orgDNA (Figure 3): (1) 5′-to-3′ exonuclease digestion of a double-stranded DNA (dsDNA) end to create a 3′ single-strand overhang; (2) binding of a single-strand annealing protein (SSAP) to this single-stranded DNA (ssDNA) region; and (3) either annealing to a homologous DNA region of another 3′-overhang end to form a concatemer or annealing of the 3′-overhang to a ssDNA gap to form a branched structure that can prime DNA synthesis and create a replication fork. ICP8 has been identified as the SSAP in HSV and possesses helix-destabilizing activity (to unwind duplex DNA), binds non-specifically to ssDNA, promotes annealing of homologous ssDNA sequences, and forms thin helical filaments and oligomeric rings in the presence of ssDNA. Is there a plastid (and mitochondrial) protein with similar characteristics to function as a SSAP? Among the organellar DNA-binding proteins that have been identified thus far (Dickey et al., 2013; Moriyama and Sato, 2014), we suggest that the Whirly family of single-strand binding proteins are good candidates to fulfill this role. Although initially implicated in the regulation of nuclear transcription and maintenance of nuclear telomeres, localization to plastids has been demonstrated for Why1 and Why3 and to mitochondria for Why2 (Marechal and Brisson, 2010). The Whirlies are DNA-binding proteins that have a higher binding affinity for ssDNA (with no sequence specificity) than dsDNA, but do promote unwinding of the ends of dsDNA (Cappadocia et al., 2010). The Whirlies form tetramers on short stretches of ssDNA and filaments on long stretches of ssDNA by cooperative binding of hexamers-of-tetramers (24-mers; Cappadocia et al., 2012). Thus Whirlies share many characteristics with ICP8 of HSV. Studies of *whirly* mutants have shown rearrangements of orgDNA likely facilitated by microhomology-mediated recombination (MHMR; Cappadocia et al., 2010; Zampini et al., 2015) and indicated that these proteins are important for maintaining organellar genome stability (Marechal and Brisson, 2010). We suggest that the filamentous Whirly-ssDNA structure ensures proper alignment of a strand-annealing end with its homologous target region and prevents MHMR as proposed for non-homologous end-joining whereby filament-forming proteins help align ends during double-strand break repair (Reid et al., 2015).

Additional functions proposed for the plant-specific Whirly protein family include attachment of plastid nucleoids to the thylakoid membrane and redox sensing in plastid-to-nucleus signaling (Foyer et al., 2014). We suggest that single-strand-binding proteins such as Whirlies also protect linear orgDNA molecules in a manner that changes during plant development. The ends of linear DNAs are susceptible to nuclease digestion unless protected by end structures including 5′-proteins, hairpin forms, and telomeric repeat sequences (Nosek et al., 2006; Chaconas and Kobryn, 2010; Smith and Keeling, 2013) and, as detailed above, the integrity of orgDNA declines sharply as maize leaves green. In yeast mitochondria the nucleoid protein mtTBP has been shown to bind to single-stranded DNA at the telomeres and has been proposed to function in the replication, stabilization, and maintenance of linear mtDNA molecules (Tomaska et al., 2001). We propose that in plastids, Whirlies bind to and protect the ends of ptDNA, as well as mediating the attachment of nucleoids to membranes. If the Whirly interaction with the membrane is responsive to the plastid redox state, then dissociation of Whirlies from the membrane and from the ptDNA ends may be triggered in photosynthetically active chloroplasts, thus releasing DNA from the nucleoid and exposing the ends to nuclease activity.

## Repair of Organellar DNA Damage

DNA damage and repair are typically studied by treating plants, animals, or their cultured cells with agents known to cause DNA damage (irradiation or peroxide, for example) and then comparing results from the treated and untreated samples (Yakes and Van Houten, 1997; Parent et al., 2011). Whereas this approach provides information about the types of DNA damage and repair processes, it provides no information about the frequency of damage/repair during the normal process of development without the imposition of genotoxic agents. It also reports the net result of damage plus repair. Another approach is to quantify the amount of transcripts, protein, or enzymatic activity from DNA-repair genes, which provides information concerning the *capacity* to repair damage, rather than the act of repair itself. For plants, some types of orgDNA lesions and repair pathways have been identified (Marechal and Brisson, 2010; Balestrazzi et al., 2011; Boesch et al., 2011; Alexeyev et al., 2013), but quantification of damage and repair as the plant develops from meristem to mature organ is only beginning to be investigated.

A common approach to study replication in the absence of repair, and *vice versa*, is to obtain mutants in one or the other component of DNA maintenance. In *Arabidopsis*, mutation in the nucleus-encoded, plastid-targeted *recA1* (*cprecA*) gene led to no alteration in leaf morphology for three generations and only a rather subtle change in leaf variegation (yellow and white sectors) in the following 4 to 8 generations—a surprisingly mild defect considering that RecA is the most highly conserved recombination protein (Rowan et al., 2010). Similarly, *Arabidopsis* single mutants of *why1* and *why3* and the double mutant *reca1polIb* resulted in no phenotypic alteration, and it was only in the *why1why3* double mutant and triple mutants *why1why3polb* and *why1why3reca1* that a defect in leaf morphology was evident (Marechal et al., 2009; Zampini et al., 2015). Thus, it appears that *Arabidopsis* employs several biochemical pathways to maintain sufficient levels of high-integrity ptDNA for chloroplast biogenesis. There was, however, a decrease in the amount of ptDNA in the *recA1*, *polIa*, and *polIb* single mutants compared to wild-type young seedlings (Rowan et al., 2010; Parent et al., 2011). Furthermore, these *recA*, *polI*, and *why* mutants exhibited alterations in ptDNA structure, including a decrease in complex replicative forms as seen by DNA Movies, loss of the monomer and oligomer bands on PFGE, and an increase in microhomology-mediated DNA rearrangements as determined by PCR and next-generation sequencing (Rowan et al., 2010; Parent et al., 2011; Zampini et al., 2015). The general conclusion in these studies was that the wild-type proteins maintain genome stability/integrity by *repair* of orgDNA. These mutations may also have disrupted the normal *replication* process by inhibiting precise recombination at defined regions (adjacent to the *oris*) that leads to branched multigenomic molecules because these proteins likely function in both replication and repair.

Since both photosynthesis and respiration produce ROS as unavoidable by-products, it may be expected that damage to orgDNA would increase as maize leaves develop. The amount of damage (measured as impediments to DNA polymerase per 10 kb of orgDNA) was lowest at the base of the stalk and increased during leaf development in the dark as well as after transfer of dark-grown seedlings to light (Kumar et al., 2014). Treatment with a mixture of enzymes that can rectify most types of DNA lesions resulted in an increase in the amount of long-PCR product for both ptDNA and mtDNA, indicating that lesions were repaired *in vitro*. Repair was much greater for leaf than for stalk tissues in both light and dark growth conditions, suggesting that orgDNA damage accumulates during “normal” growth conditions (without genotoxic treatment) without causing phenotypic change.

To summarize, although the capacity to repair damaged orgDNA has long been known for plants and animals, only recently—and for only one plant species—has repairable damage of orgDNA been quantified under normal development without the addition of stress or genotoxic agents. Light affects both damage and levels of functional DNA in both plastids and mitochondria, even though mitochondria have no known photoreceptors. Most “copies” of orgDNA from normal light-grown plants that are measured by standard qPCR are too highly degraded to serve a coding function, at least for maize. Although this conclusion likely applies to *Arabidopsis* (Rowan and Bendich, 2009), we currently lack long-PCR and *in vitro* repair assay data in order to evaluate the quantity and quality of orgDNA molecules as proplastids (and their mitochondrial counterparts) mature to the organelles found in the green leaf. New insight may be anticipated once the replication/repair mutants of *Arabidopsis* are identified in maize so as to complement the advantage of the linear gradient of staged cell development in maize leaves. One possibility is that repair in maize occurs only in the meristem, so that unrepaired orgDNA in the green chloroplasts is degraded: orgDNA abandonment.

## Differences in Leaf Growth, Plastid Development, and Organellar DNA Maintenance Among Plant Species

**FIGURE 4:**
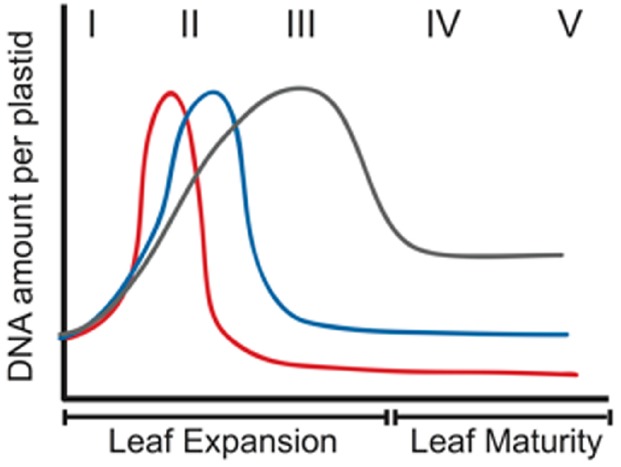
**Schematic representation of changes in the amount of ptDNA per plastid during development in three plant species.** Increase in ptDNA amount due to ptDNA replication occurs very early in development in maize (red line), followed by a rapid decline. For *Arabidopsis* (blue line), the increase in ptDNA occurs slightly later and the decline in ptDNA amount is much later. For tobacco (gray line), ptDNA increases more gradually and the decline is less severe. The Roman numerals indicate stages of leaf development. I–III represent expanding leaves, and IV and V represent expanded leaves. (Reprinted from Rowan and Bendich, 2009).

During proplastid-to-chloroplast development, the DNA level per plastid first increases and then decreases, although the magnitude of the decline varies among species. For example, ptDNA increases later and remains high longer for both *Arabidopsis* and tobacco than maize (Figure 4). In mature tobacco leaves, nearly all cells contain chloroplasts with DAPI-fluorescent nucleoids (Shaver et al., 2006), whereas nucleoids are not detectable in ≥40% of maize cells (Oldenburg et al., 2014). Furthermore, the genomic monomer and oligomers are prominent in PFGE of ptDNA from mature leaves of many dicots, but in maize even the monomer is barely detectable (Lilly et al., 2001; Oldenburg et al., 2006; Shaver et al., 2006). These differences in ptDNA maintenance may result from differences in leaf growth and ptDNA-associated proteins.

Leaves of grasses, such as maize, exhibit a base-tip developmental gradient: dividing cells are restricted to the basal meristem; developing and elongating non-photosynthetic cells in the stalk are shielded from light by the coleoptile and/or outer sheath; and the mature leaf blade consists of fully-differentiated photosynthetic cells (Nelson and Langdale, 1989; Sylvester et al., 1990; Tardieu and Granier, 2000; Stern et al., 2004). In dicots, such as *Arabidopsis* and tobacco, cell division is not restricted to the apical meristem, but continues along a base-to-tip gradient in the expanding leaf (Donnelly et al., 1999; Tardieu and Granier, 2000; Rowan and Bendich, 2009). Except for the meristem, which is enclosed in the bud and shielded from light, cell development and elongation occur in the light. Thus in grasses, there is a prolonged etioplast-like developmental stage in the expanding leaf followed by an abrupt transition to a green chloroplast as the leaf tip emerges from the sheath, whereas photosynthetic chloroplasts are present throughout development of a dicot leaf. The ROS produced during photosynthesis would necessitate greater ptDNA-protective measures in the expanding dicot leaf, which could persist (at a reduced level) in mature leaves. In contrast, little ptDNA protection is evidently provided in green chloroplasts of maize, as indicated by the rapid ptDNA decline upon light exposure (Zheng et al., 2011). There are also differences in DNA maintenance proteins. For example, *Arabidopsis* has two DNA polymerases, PolIA and PolIB, with PolB implicated in ptDNA repair (Mori et al., 2005; Parent et al., 2011), whereas only PolIA has been reported for maize (Majeran et al., 2012; Udy et al., 2012). In maize only one Whirly protein, Why1, has been reported (Marechal et al., 2009; Majeran et al., 2012), whereas both Why1 and Why3 are present in *Arabidopsis* (Marechal et al., 2009; Cappadocia et al., 2010) where Why3 could provide protection against nucleases in chloroplasts by mediating DNA-nucleoid-membrane attachment. Therefore, greening during the etioplast-to-chloroplast transition in maize would lead to loss of ptDNA from ROS-mediated damage without repair. In *Arabidopsis* and other dicots, when the level of ptDNA damage exceeds the protective/repair capacity, ptDNA would also be degraded, although this would occur later in leaf development (Figure 4).

These dicot/grass differences in ptDNA maintenance may have ecological and evolutionary ramifications. The ptDNA in the dicot leaf must be kept in good repair—and at substantial cost—during the period of ptDNA replication, which is concurrent with photosynthesis and chloroplast expansion. In grasses, by contrast, etioplast expansion to a size equivalent to a green chloroplast, ptDNA replication, and, critically, production of all the ptDNA-encoded mRNAs required for photosynthesis during the coming plant growth season, all proceed without the DNA-damaging ROS by-product of photosynthesis. The ptDNA may, therefore, be abandoned in green chloroplasts, avoiding the metabolic cost of ptDNA repair. Thus, leaf ptDNA maintenance is “low-cost” in the grass and “high-cost” in the dicot leaf.

This cost saving may have contributed to the rapid rise of grasses beginning in the Late Cretaceous-Paleocene (Strömberg, 2011; Christin et al. (2014)). Replacement of the ancestral apical meristem proplastid-to-chloroplast progression in dicots with a basal meristem proplastid-to-etioplast-to-chloroplast transition in grasses may have been advantageous. In mid-latitudes 55–70 million years ago, selective pressures included seasonally dry climates, wildfires, and herbivory (Bond and Scott, 2010). A ground-level basal meristem may provide greater tolerance to drought-stress and defoliation by mammals. By abandoning ptDNA in mature leaves, grasses may realize a cost saving by not repairing DNA damaged by increased ROS from drought-stress and not investing in ptDNA maintenance in mature leaves that would be lost to fire or herbivory.

## Can Organellar DNA Really be Lost in Healthy Leaves?

We have a relatively good understanding of the replication and repair apparatus that maintains nuclear DNA at a constant, diploid level throughout development. By comparison, there is disagreement concerning the maintenance of orgDNA in the same cells. Rather than infer the properties of orgDNA molecules from enzyme requirements and indirect methods like RNA analysis, the quality, quantity and stability of orgDNA molecules themselves should be investigated during development from meristem to green leaf.

The data showing the demise of orgDNA during leaf maturation have not been well received by some, and the controversy has been presented recently (Golczyk et al., 2014; Oldenburg et al., 2014). There are four main reasons for skepticism. First, some proteins, especially the product of the *psbA* gene (D1), turn over very rapidly and must be continuously replaced for photosynthesis to occur. Thus, either there must be a functional *psbA* gene in the green chloroplast to supply the mRNA for ongoing production of D1 protein during photosynthesis or the mRNA for D1 is extremely stable. In dismissing the latter alterative, the half-life of *psbA* mRNA (for barley) was mistakenly cited as “in the range of 40 h” (Golczyk et al., 2014), whereas the reported half-life was >40 h, the mRNA level did not change over a 30-h period, and mRNA stability increased during chloroplast development (Kim et al., 1993).

The second reason for skepticism is the fact that ptDNA copy number estimated from standard qPCR is ∼800 to 1400 copies per haploid nuclear genome in mature green leaves of maize, with the assumption that each copy measured from a 0.15-kb PCR product represents a genome-sized molecule. Although the same approximate number was reported by both parties to the controversy, data from DNA Movies and PFGE and, more recently, from miPCR indicated that essentially all of those “copies” were present as highly-fragmented or lesion-containing ptDNA molecules, as discussed above.

The third reason is that an *in vitro* run-on transcription assay shows that ptDNA is present in the chloroplasts isolated from green leaves of barley (Emanuel et al., 2004). In this assay, radiolabeled UTP is incorporated into the growing RNA chain that had been initiated before the leaves were harvested. However, the fraction of the millions of chloroplasts in the assay tube that are engaged in transcription is unknown—it could be <1 or 100%—and rare proplastids in the chloroplast preparation could be the source of the transcription activity. Furthermore, transcripts from highly-fragmented ptDNA might not benefit the cell from their coding potential, but instead represent the residuum from transcription-coupled repair, a proposed global surveyor of DNA damage (Epshtein et al., 2014) and suggested to occur early in the development of plastids and mitochondria (Kumar et al., 2014).

The suggestion has also been made that the data indicating the demise of orgDNA are due to methodological artifacts (Golczyk et al., 2014; disputed by Oldenburg et al., 2014). Furthermore, for the artifact alternative to be correct, each of the types of data that document the decline of orgDNA—PFGE, DNA Movies, quantitative DAPI fluorescence, and miPCR—would have to be affected by an independent artifact, with none of these hypothetical artifacts occurring when we studied the orgDNA from the meristematic tissue. We conclude that during proplastid-to-chloroplast development, the ptDNA level initially increases to supply the gene products needed for photosynthesis. After chloroplast maturation, excess copies are no longer needed, degraded, and the nucleotides recycled. The net result is a decrease to a low constant ptDNA level in mature leaves with many molecules too damaged or fragmented to serve a coding function, even if they can be scored as “genome copies” by qPCR.

The fourth reason is that cytological images of DAPI-stained nucleoids indicate the persistence of some ptDNA in expanded green leaves of several plants (Golczyk et al., 2014). These data, however, are not quantitative, do not reflect the quality of the ptDNA molecules, and do not report the fraction of DAPI-positive chloroplasts among chloroplasts chosen at random for analysis. The genome copy number per individual chloroplast chosen at random before quantitative analysis of DAPI fluorescence varied from 0 to 241 for the first green leaf of maize (Zheng et al., 2011; Oldenburg et al., 2014); 0 to 82 for the mature first rosette leaf of *Arabidopsis* (Rowan et al., 2009); 6 to 259 for the mature 16th leaf of tobacco; and 0 to 194 for the fully-expanded second leaf of *Medicago trunctatula* (Shaver et al., 2006). In each case, DNA Movies showed that the ptDNA was highly fragmented. Thus the detection of DAPI-positive nucleoids does not necessarily indicate that the nucleoids contain ptDNA molecules of high quality.

## Concluding Remarks

The amount and degree of molecular integrity of DNA present in a particular tissue are determined by replication, repair, and stability of the DNA. For the diploid nucleus, these processes are governed by checkpoint control in such a way as to result in a constant amount of stable, intact chromosomal DNA molecules throughout development, regardless of the physiological activities of the cells. For plastids and mitochondria, however, such tight control is not exercised, and the amount and quality of orgDNA can vary greatly among tissues, from pristine multigenomic chromosomes in meristematic cells to highly fragmented “copies” in mature leaves, without compromising the homeostasis of the wild-type plant. In other words, orgDNA—but usually not nuclear DNA—can be abandoned in somatic cells as part of the normal developmental process. In the single-celled alga *Euglena*, orgDNA cannot be abandoned but ptDNA and mtDNA are unstable (half-lives of 1.6 and 1.8 cell doublings, respectively), whereas nuclear DNA turnover could not be detected (Manning and Richards, 1972; Richards and Ryan, 1974). The advantage of DNA abandonment leading to DNA-repair cost savings and embryonic development in plants and animals has been discussed previously (Bendich, 2010b, 2013). Although DNA could not be abandoned in the bacterial ancestors of plastids and mitochondria, orgDNA abandonment in leaves has evidently been advantageous, especially for grasses.

### Conflict of Interest Statement

The authors declare that the research was conducted in the absence of any commercial or financial relationships that could be construed as a potential conflict of interest.
